# Essential Oils from Bolivia. XV. Herzogole, an Original Monoterpene Benzodioxole from an Essential Oil from *Pentacalia herzogii* (Cabrera) Cuatrec

**DOI:** 10.3390/molecules26195766

**Published:** 2021-09-23

**Authors:** Alexis St-Gelais, Eliana M. Maldonado, Gloria Saavedra, Samuel Siles-Alvarado, Jérôme Alsarraf, Guy Collin, André Pichette

**Affiliations:** 1 Laboratoire Phytochemia, 628, Boulevard du Saguenay Ouest, Saguenay, QC G7J 1H4, Canada; 2 Centro de Tecnología Agroindustrial, Universidad Mayor de San Simón, Cochabamba 992, Bolivia; elianamaldonado.g@fcyt.umss.edu.bo (E.M.M.); gloriasa@fcyt.umss.edu.bo (G.S.); 3 Centro Especializado de Instrumentación Analítica, Universidad Mayor de San Simón, Cochabamba 992, Bolivia; samuelsiles@fcyt.umss.edu.bo; 4 Laboratoire d’Analyse et de Séparation des Essences Végétales (LASEVE), Centre de Recherche sur la Boréalie (CREB), Département des Sciences Fondamentales, Université du Québec à Chicoutimi, Saguenay, QC G7H 2B1, Canada; jerome1_alsarraf@uqac.ca (J.A.); Guy_Collin@uqac.ca (G.C.); andre_pichette@uqac.ca (A.P.)

**Keywords:** *Pentacalia herzogii*, *Senecio* sp., essential oil composition, isothymol methyl ether, thymol methyl ether, α-pinene, herzogole, nuclear magnetic resonance (NMR)

## Abstract

Over 15 years, with the support of a Canadian funding agency, the Universidad Mayor de San Simón, in Bolivia, undertook a large survey of aromatic plants of the South American country. More than a hundred species were studied under various aspects, including the production and characterization of essential oils. As part of this survey, the chemical composition of an essential oil sample obtained from *Pentacalia herzogii* (Asteraceae) growing wild in the High Valley region of the department of Cochabamba was determined by a combination of GC and GC-MS measurements. α-Pinene was the main constituent of this essential oil (34%), accompanied by limonene (22%) and germacrene D (7.5%) as well as an important fraction of methoxylated monoterpenoids. They were mainly isomers of thymol methyl ether, accounting for 13% of the chromatogram. A new quantitatively important compound (9%) was identified through NMR and chemical synthesis as 4-isopropyl-6-methylbenzo[*d*][1,3]dioxole, and designated herzogole, alongside the minor related compound 1-isopropyl-2,3-dimethoxy-5-methylbenzene. The monoterpene benzodioxole featured a distinctive green-phenolic aroma which could raise interest for fragrance use. Since these compounds were not known naturally, a biosynthetic mechanism of their formation was proposed and put in perspective to illustrate the metabolic originality of *P. herzogii*.

## 1. Introduction

In the late 1980s until the early 2000s, several research groups of the Universidad Mayor de San Simón, Cochabamba, Bolivia, have driven a large survey of the Bolivian flora. This effort was conducted with the support of the International Development Research Centre (IDRC) in Canada. In this regard, agronomists, biochemists, phytochemists, engineers and chemists joined efforts to gather knowledge about national plant species. One of the objectives of this multidisciplinary endeavor was to identify potential crops and vegetable resources that could in part replace the traditional coca plantations in Bolivia and provide revenue to local populations.

Within the phytochemical investigation efforts, one group was particularly devoted to the examination of essential oils. Given their expertise in the field, partners from the Université du Québec à Chicoutimi, Québec, Canada, joined the project to provide support with training, techniques and determination of potential commercial outcomes. During the course of the program, over 100 aromatic plant samples were sampled and sorted in Bolivia, and about 70 were studied for their essential oil content and characteristics. Of course, not all of those gave conclusive results, with several yielding too little essential oil to warrant further examination. A few other plants were not botanically identified at the time, such as *Clinopodium axillare*, for which ensuing disambiguation [1] permitted publication of the essential oil composition more recently [2]. This latter publication continued a series of 20 previous papers reporting some of the most interesting findings of this essential oil survey. Among them, a selection of the most relevant or important is listed below [3,4,5,6,7]. The biological evaluation of 20 of the Bolivian essential oils tested showed some insecticidal activity against the vector of Chaga’s disease, an important illness in several regions spanning from the Peruvian highlands to the dry northeast Brazil [8]. Several of those plants also showed interesting antioxidant potential, evaluated using the β-carotene bleaching technique [9].

Among the other essential oils screened, owing to the presence of a major unknown constituent (≈15% of the chromatogram [10]) alongside limited availability of the sample, the results for the essential oil composition of *Pentacalia herzogii* (Cabrera) Cuatrec. were up to now left aside. This Asteraceae species, formerly known as *Senecio herzogii*, is a scandent aromatic shrub locally known as “falsa mora” and is mainly found in Bolivia [11]. The woody vine is approximately 1 to 1.8 m in length. The stems are terete, longitudinal furrows, with arachnoid-flucosse indumentum in terminal buds of young branches. The alternate leaves, with barely dentate margins, are oblong-elliptic with an obtuse apex, glabrous on the surface and lanate on the underside. The synflorescence is mostly terminal, broadly paniculiform-thyrsoid in shape, with disciform and sessile capitula clustered at the distal part of each lateral synflorescence. The florets are five-lobed and tubular. To the best of our knowledge, no previous phytochemical study was conducted for this species. Nevertheless, the preliminary results had shown interesting essential oil yield for this species, alongside a distinctive, phenolic-green aroma for the distillate. This warranted reexamination of a *P. herzogii* essential oil sample distilled from a contemporary plant collection, supplying enough distillate to allow for characterization of the major unknown constituent therein, as a solid basis for a potential future valorization of the species. We hereby report the isolation and characterization of this new compound, alongside the detailed analysis of a sample of *P. herzogii* essential oil.

## 2. Results and Discussion

The composition of the *P. herzogii* essential oil sample appears in Table 1. This batch of essential oil is characterized by a high percentage of α-pinene (33.6%), limonene (21.6%) and germacrene D (7.5%). More interestingly, three oxygenated monoterpenes with molecular mass of 164 amu account for 13.2% of the essential oil. They are three isomers, namely 1-methoxy-4-methyl-2-(1-methylethyl)-benzene, 2-methoxy-4-methyl-1-(1-methylethyl)-benzene and 2-methoxy-1-methyl-4-(1-methylethyl)-benzene, more commonly known as isothymol, thymol and carvacrol methyl ethers, respectively [12]. The first two molecules account for about the same percentages (6.0% and 6.6%). The carvacrol methyl ether percentage is less than 1%. Overall, this profile corresponds well with the preliminary observations on a previous *P. herzogii* essential oil [10], which featured 32% α-pinene, 12% isothymol methyl ether, 11% thymol methyl ether and 8% germacrene D; limonene was, however, not found in significant proportions back then (0.5%).

In the Asteraceae family, the genus *Senecio* sensu lato occupies an important place (more than 1500 species). Despite this great diversity, only about one hundred and sixty papers devoted to the study of the essential oils and volatile compounds of *Senecio* species (which was the former genus of *P. herzogii*) are reported in the SciFinder^®^ database [13]. Less than ten of them show the presence of low percentages (<2%) of thymol methyl ether and only three the occurrence of carvacrol methyl ether (Table 2) [14,15,16,17,18,19,20,21,22]. Surprisingly, one paper mentions the ambiguous *o*-methyl thymol as an identified compound in the *S. scandens* essential oil [19]. In South America, the reported studies indicate that hydrogenated monoterpenes constitute the most important part of the identified compounds in *Senecio* essential oils. For example, this is the case of essential oils obtained from Argentina [23,24], Chile [25] and Peru [26]. Three members of this genus *Senecio* stand apart. Essential oil of the leaves *S. crassiflora* from Brazil shows the presence of two quantitatively important sesquiterpenols, τ-muurolol and α-cadinol. Germacrene D is the most important compound of the stem of the same species [27]. Another hydrogen sesquiterpene, α-zingiberene, is also the main constituent of the *S. selloi* essential oil from Brazil [28]. Dehydrofukinone is by far the most important compound observed in the case of *S. viridis* from Argentina, since it constitutes more than 92% of the essential oil [29].

This comparison with other closely related species of Asteraceae suggests that the *P. herzogii* specimens that yielded this essential oil were phytochemically original, based on the high proportions of monoterpenic ethers. Thymol or isothymol methyl ethers as a proportionally important essential oil constituent are more commonly encountered in Lamiaceae species, e.g., *Origanum* [30,31] and *Thymus* [32], or Apiaceae species, such as *Cyclospermum* [33], *Ammoides* [34] and *Apium* [35]. In these cases, the methyl ethers accumulate in conjunction with thymol, carvacrol or thymoquinone dimethyl ether. Another pattern is found in the Apiaceae *Crithmum maritimum* [36,37,38,39], where important proportions of thymol methyl ether correlate with large amounts of γ-terpinene, a direct metabolic precursor of thymol [40]. Of these four biosynthetically correlated constituents, only small amounts of γ-terpinene and traces of carvacrol are encountered in the studied *P. herzogii* essential oil accession, further highlighting the metabolic originality of those individuals among aromatic plants owing to their ability to accumulate methyl ethers selectively.

The percentage for this essential oil of a fourth and until now unidentified compound **1** is 9.2%, and its structural elucidation was one of the driving interests for the present study. Indeed, thoroughly characterizing essential oils is important from both a theoretical and practical perspective. On the one hand, it is an important endeavor to keep on expanding the knowledge assembled over decades of research on essential oils in an effort to characterize both major and minor compounds, which can then in turn refine the comprehension of biosynthetic routes involved or allow for easier analysis of other species later on (e.g., [41]). On the other hand, the quality control of essential oils relies on knowledge of both quantitatively important compounds but also minor signature compounds, and the continued expansion of the trade of essential oils worldwide calls for precise and thorough analyses, which in turn rely on expanding theoretical knowledge.

**Figure 1:**
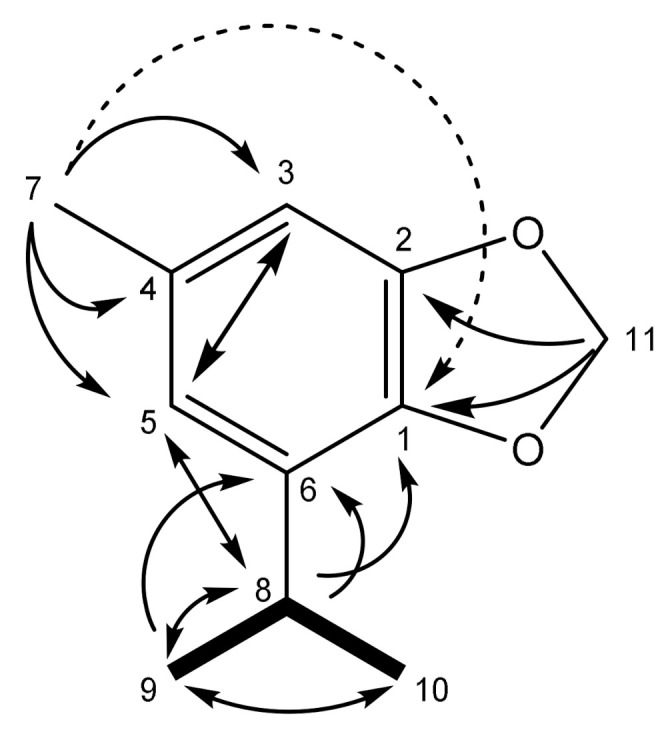
Structure of herzogole **1** with DQF-COSY correlations in bold bonds and HMBC correlations (H→C) indicated by arrows. An unexpected correlation is shown by a dashed arrow (see text).

**Figure 2:**
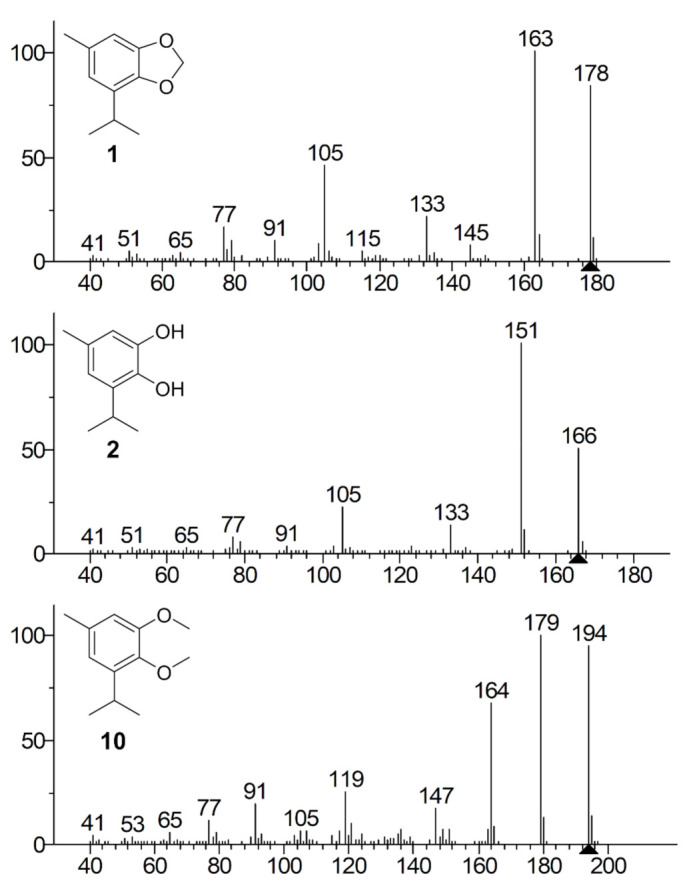
Mass spectra of compounds **1**, **2** and **10**.

This unknown compound has a very similar mass spectrum to those of the three methoxy ethers just identified above, and it was hypothesized that it bore a metabolic relationship to the latter. The main difference lies in the fact that its main ions are 14 uma units higher. This value could have suggested the presence of a CH_2_ group added to one of the three methoxy ethers. Taking thymol methyl ether as a reference, the unidentified compound has a retention index of about 100 units higher on the non-polar column, in agreement with the known additive rule for the retention indices [42,43]. However, this difference in the polar column is about 200 units higher and then does not follow the same additive rule, ruling out the ethoxyl substituent hypothesis. A sample of essential oil was therefore submitted to column chromatography to afford a fraction of pure compound **1** as a clear liquid. ^1^D NMR experiments suggested the presence of an aromatic ring, three methyls, one methylene and one methine. Methylene C-11 was notably deshielded on both ^1^H and ^13^C spectra, with shifts characteristic of acetal groups. This, along with the HMBC correlations of H-11 with aromatic C-1 and C-2 (Figure 1), indicated that the compound featured a benzodioxole moiety. A spin system comprising H-8 and the equivalent methyls H-9 and H-10 was visible on the DQF-COSY spectrum, with multiplicities characteristic of an isopropyl substituent. The HMBC cross-peak between H-8 and aromatic methine C-5 and quaternary aromatics C-1 and C-6 indicated that this substituent was vicinal to one of the methylenedioxy groups and that the other *ortho* position was unsubstituted. Another methyl singlet, H-7, was also observed on the ^1^H spectrum, with clear HMBC correlations to quaternary C-4 and aromatic methines C-5 and C-3, suggesting that both *ortho* positions were unsubstituted. Therefore, the observed spectroscopic data were reasonably consistent with the structure of 4-isopropyl-6-methylbenzo[*d*][1,3]dioxole **1**. Its mass spectrum is featured for reference in Figure 2.

**Scheme 1:**
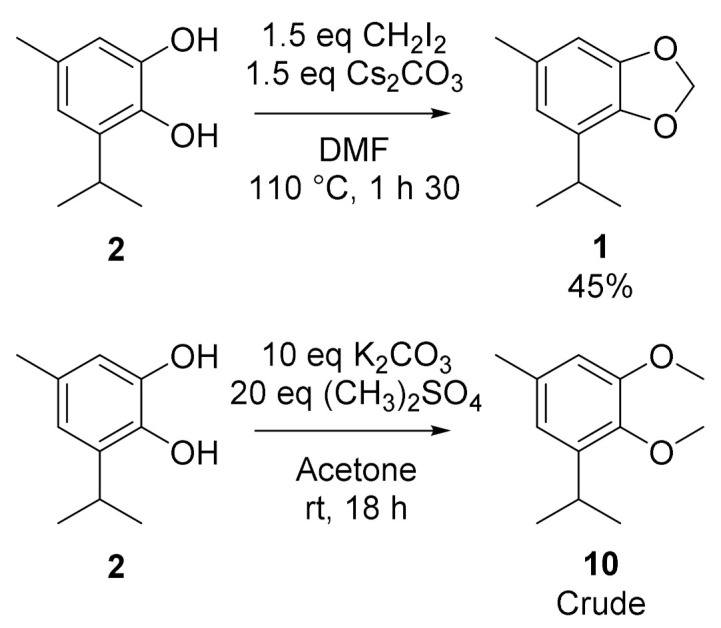
Preparation of herzegole **1** and 1-isopropyl-2,3-dimethoxy-5-methylbenzene **10** via their precursor catechol **2**.

However, an unexpected HMBC cross-peak was found between methyl H-7 and the methylenedioxy attachment point C-1, beyond typical correlations expected. Furthermore, ^1^H peaks for H-5 and H-3 exhibited the same chemical shift, appearing as an indistinct singlet that did not allow the observation the characteristic *meta* 2-3 Hz coupling constant that would have fully validated the proposed structure. These peculiarities prompted further investigation. The putative structure of **1** was therefore confirmed through chemical synthesis. Commercial 5-methyl-3-(propan-2-yl)benzene-1,2-diol **2** was reacted with diiodomethane in the presence of cesium carbonate in hot dimethylformamide [44], affording compound **1** (Scheme 1). Superimposition of ^1^H and ^13^C spectra of the isolated and synthesized compounds confirmed that both molecules were identical and that the proposed structure was correct, despite the unusual HMBC correlation. Examination of the observed fragmentation pattern was also consistent with the structure (see Supplementary Materials). By analogy with safrole, another molecule with a benzodioxole nucleus, the compound was given the trivial name herzogole.

**Scheme 2:**
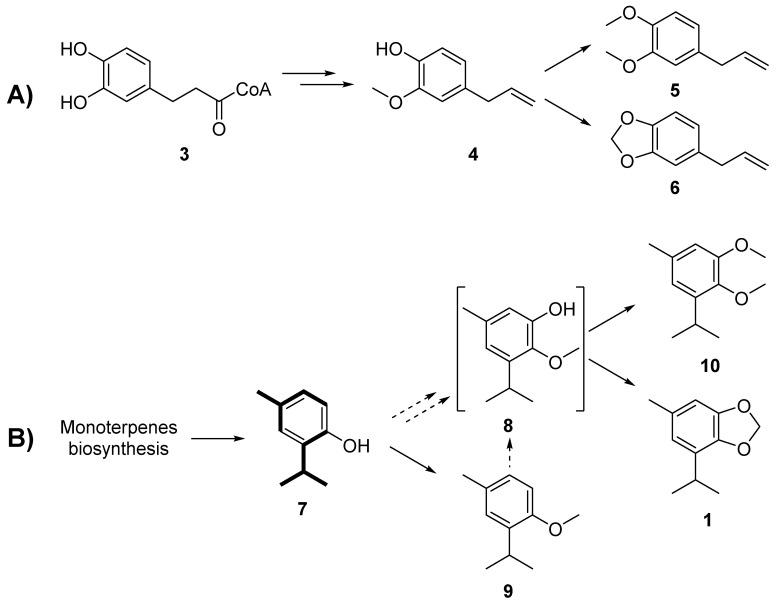
(**A**) Proposed biosynthetic route of methyleugenol **5** and safrole **6** in *Asarum sieboldii* [45]. (**B**) By analogy, hypothetical biosynthetic relationships of isothymol **7** (the two isoprene units from monoterpenes are shown in bold in isothymol), with isothymol methyl ether **9**, a putative intermediate compound **8**, isolated herzogole **1** and a biosynthetic dimethylated analogue **10**.

The presence of a benzodioxole moiety in a monoterpenoid is somewhat surprising, as this moiety is more characteristic of phenylpropanoids. To our knowledge, this compound has never been mentioned in the literature (no match in the SciFinder® database [13]). Thus, the question of its origin must be discussed to discard any possibility of an artifactual observation. The GC-MS analysis carried out in 1998 on a preliminary sample indicated its presence in a higher concentration, at about 15% of the essential oil [10]. The use of any undisclosed biocide in this almost virgin region is highly improbable, especially 20 years apart and in such high proportions of the essential oil. These observations lead us to believe that this unknown compound is not of exogenous origin. Conversely, by analogy with the proposed biosynthesis from caffeoyl coenzyme A (**3**) to eugenol (**4**) which is then converted either to methyleugenol (**5**) or safrole (**6**) in *Asarum sieboldii* [45], a plausible biosynthetic tree can be proposed from isothymol **7**, arising from the monoterpene biosynthesis (Scheme 2). The existence of a terpenophenol *O*-methyltransferase is apparent in *P. herzogii* given the presence of abundant thymol methyl ether, carvacrol methyl ether and isothymol methyl ether **9** in the essential oil. Isothymol **7** or the methyl ether **9** could also be hypothetically converted to the monomethylated intermediate **8** (or its isomer where the methyl is bonded to the other oxygen), not readily observed in the essential oil. The latter could then either proceed towards herzogole **1** by the action of a cytochrome P450 or be further methylated by an *O*-methyltransferase to generate 1-isopropyl-2,3-dimethoxy-5-methylbenzene **10** (which was only reported once in the literature as a synthesis intermediate [46]), as for the biosynthetic trade-off that exists between safrole **6** and methyleugenol **5**. Compound **10**, with a mass of 194 amu, was interestingly consistent with a small unknown compound observed at RI_DB-5_ = 1387, whose mass spectrum showed not only this mass but also clear *m/z* at −15 and −30 amu, consistent with the abduction of one or two methyls (Figure 2). To verify whether compound **10** was indeed present in the essential oil, it was also prepared from a small remaining amount of catechol **2**, using excess dimethyl sulfate to methylate both hydroxyls. Although an insufficient amount was obtained to attempt purification and characterize the obtained yield, comparison of the NMR of the crude product to published data [46] clearly showed the formation of the desired compound as the main reaction product. This allowed us to establish the identity of **10** by direct comparison with the main GC peak of this crude product. The simultaneous presence of isothymol methyl ether **9**, herzogole **1** and compound **10** supports the plausibility of the proposed biosynthetic route (Scheme 2), which serves as an argument in favor of the endogenous nature of herzogole within the distilled accession of *P. herzogii*. This yet unique biosynthetic pattern leading to accumulation of a rare monoterpenic benzodioxole further stresses the metabolic originality of this species’ sample and illustrates how the examination of previously unstudied plants can uncover new aspects of phytochemistry.

The above synthesis of herzogole allowed us to confirm another property of compound **1**. The isolated product has a strong green-phenolic scent, like that of the whole *P. herzogii* essential oil obtained in the course of this study. One could not rule out, however, that a minute impurity with high aroma potency from the essential oil would induce this scent. The synthesized product featured the same aroma, while being unlikely to contain the same hypothetical impurity as the essential oil-derived purified fraction. This suggests with some confidence that herzogole is one of the major aroma contributors of the *P. herzogii* essential oil and could by itself bear some potential valorization as a fragrant compound. Otherwise, a broader screening of *P. herzogii*, which is encountered as scattered individuals in the wild, should be conducted in the future to further assess potential variability of this metabolic pathway following, e.g., environmental or genetic factors.

Although sesquiterpenes, to the exception of germacrene D, are not prominent constituents of the tested essential oil, some minor compounds need a brief justification of their identification. Both gynurenol and gynuradienol are rather rare compounds. They were identified as volatile constituents from the roots of *Gynura bicolor* DC, another plant belonging to the Asteraceae family. Their retention indexes on both non-polar and polar columns agree with the published values. The same is true for the recorded mass spectra [47]. 4,10-*Diepi*-guaiol was identified by comparison with an analysis of *Boswellia occulta* essential oil on the same chromatographic system, following published results [48]. Isoguaiols are also unusual compounds. They were described through their ^1^H-NMR, ^13^C-NMR and mass spectra. Their retention indices on both polar and non-polar columns were also published [41]. In fact, of eight possible optical isomers, four were characterized, namely isoguaiol A, B, C and D: (1β*H*,7α*H*,10α*H*)-, (1α*H*,7α*H*,10β*H*)-, (1β*H*,7α*H*,10β*H*)- and (1α*H*,7α*H*,10α*H*)-guai-4-en-11-ol, respectively; they all feature very similar mass spectra. Given this ambiguity and the differences in reported retention indexes between the two chromatographic systems, the observed peak could correspond either to one or a mixture of at least two of the available structures. Thus, the proposed identification as isoguaiol isomer(s) is not unreasonable. Bulnesoxide was tentatively identified based on the same study [41], and a liguloxide epimer also was conditionally labeled based on high mass spectral similarity, but a non-matching retention index with liguloxide itself.

## 3. Conclusions

The present study allowed us to clarify a two-decade-old unresolved result from a larger screening of Bolivian aromatic plants essential oils, conducted to provide valorization perspectives of the local flora and alternatives to the traditional coca culture. Previously, despite promising yield and aroma, an essential oil of *Pentacalia herzogii* could not be fully characterized earlier due to the presence of a major unknown constituent. Reexamination of a freshly distilled essential oil allowed us to identify herzogole, a previously unreported monoterpenic benzodioxole, as one of the main constituents of the essential oil, alongside thymol and isothymol methyl ethers. Rationalization of its probable biosynthesis and comparison with essential oils from related *Senecio* species and typical monoterpene methyl ether rich essential oils illustrate how those specimens of *P. herzogii* stand out as metabolically original among aromatic plants and allowed us to further identify a minor dimethoxylated monoterpene not previously reported in nature. Herzogole also bears a characteristic green-phenolic aroma, akin to that of the whole essential oil studied herein. This study overall allowed us to report 92 compounds of this essential oil and provided solid analytical ground for future studies and valorization of this plant in Bolivia.

## 4. Materials and Methods

### 4.1. Plant Material

At the end of the flowering period, the aerial parts of *P. herzogii* were collected along the road going from Corani-Pampa to Tablas Monte, Chapare, Bolivia (13 km from Corani, altitude: 2430 m; 17°06′11″S and 65°58′44″W) at the end of October 2019. Voucher specimens were authenticated with their morphological and anatomical features and have been deposited in the Herbario Nacional Forestal Martin Cardenas (BOLV) of the Universidad Mayor de San Simón, Cochabamba, Bolivia, under the registration code MZ 6781. Fresh leaves and stems were cut into pieces, pooled together as a single sample and placed in plastic bags and frozen at −18 °C until the oil extraction.

### 4.2. Essential Oil Extraction

A total biomass of 0.5 kg of the aerial parts (frozen leaves and stems collected together from several individuals) was hydrodistilled for 3 h in a glass distillation apparatus (FIGMAY, Argentina) set to maintain a constant level of 1.5 L of water through continuous water supply to yield 0.9% of the essential oil. Yield was calculated by dividing the mass of essential oil obtained by the mass of the frozen raw material, and multiplying by 100%. It was dried on anhydrous sodium sulfate and stored in amber glass vials at 4–5 °C until analysis. A single distillation was conducted.

### 4.3. General Experimental Procedures

The GC parameters are described in Table 3. Identification of the components was done by comparison of their retention indices (RIs) with normal alkanes from C7 to C40 and by comparison of their mass spectra with literature data [49,50,51] and with our own databases. Quantitative data were obtained electronically from GC-FID area percentages without correction (internal normalization).

NMR spectra were recorded with a Bruker Avance 400 spectrometer at 400 MHz for ^1^H nuclei and 101 MHz for ^13^C nuclei, using deuterated chloroform (CDCl_3_) as the solvent. Chemical shifts are reported in ppm relative to the solvent residual peak (δ = 7.26 ppm for ^1^H and 77.16 ppm for ^13^C). Reactions were monitored by thin-layer chromatography (TLC) with silica gel 60 F_254_ 0.25 mm pre-coated aluminum foil plates (MilliPore) and visualized under UV_254_. Chemical reactants were from Millipore Sigma (Burlington, MA, USA).

### 4.4. Isolation, Synthesis and Structural Elucidation

4-Isopropyl-6-methylbenzo[*d*][1,3]dioxole (herzogole; **1**): Crude essential oil (1.49 g) was deposited on a chromatographic column comprising 200 g of silica gel 60 (15–40 µm, Silicycle, Québec, QC) previously wetted with *n*-hexane. The sample was eluted with pure *n*-hexane, then 9:1 and 8:2 mixtures of *n*-hexane and dichloromethane to afford compound **1** as a green-phenolic-scented light-yellow liquid (27 mg), alongside more fractions of **1** contaminated with thymol and isothymol methyl ethers. Compound **1** was also prepared by suspending 5-methyl-3-(propan-2-yl)benzene-1,2-diol (**2**; 63 mg, 0.38 mmol) in 3 mL dry dimethylformamide under argon atmosphere, then adding Cs_2_CO_3_ (186 mg, 0.57 mmol) and diiodomethane (45 µL, 0.57 mmol). The solution was heated to 110 °C for one hour, cooled down, then quenched with saturated aqueous NH_4_Cl. The reaction product was extracted with 3 × 5 mL ethyl acetate, and the combined organic phases were dried over sodium sulfate and evaporated under reduced pressure. The dried residue was purified by flash chromatography over 4 g silica, eluting with 15 mL pure *n*-hexane then *n*-hexane/dichloromethane (7:3) until the product was obtained as a green-phenolic-scented clear liquid (30.6 mg, 45% yield).

RI (DB-5/ Wax): 1330/1749; Rf = 0.54 (Hex/DCM 5:1); ^1^H NMR (400 MHz, CDCl_3_) δ: 6.53 (s, 2H, H-3/H-5), 5.90 (s, 2H, H-11), 3.00 (sept, *J* = 7.0 Hz, 1H, H-8), 2.27 (s, 3H, H-7), 1.25 (d, *J* = 7.0 Hz, 6H, H-9/H-10); ^13^C NMR (100 MHz, CDCl_3_) δ: 147.2 (C-2), 142.6 (C-1), 131.4 (C-4), 129.8 (C-6), 119.7 (C-5), 107.3 (C-3), 100.6 (C-11), 29 (C-8), 22.4 (C-9/C-10), 21.5 (C-7); EI-MS (70 eV) *m/z* 178 [M]^+^ (84), 163 (100), 150 (1), 145 (7), 133 (21), 119 (3), 105 (45), 91 (10), 79 (9), 77 (16).

5-Methyl-3-(propan-2-yl)benzene-1,2-diol (**2**): The compound, of 95% purity, was purchased from Enamine LLC (Monmouth Jct., NJ) as a custom synthesis product, batch # 2017-0154152. The certificate of analysis is provided in the Supplementary Materials. Since gas chromatographic and mass spectral data do not appear to have been reported previously, they are provided for convenience.

RI (DB-5/ Wax): 1487/2728; EI-MS (70 eV) *m/z* 166 [M]^+^ (50), 151 (100), 133 (13), 123 (3), 105 (22), 103 (4), 91 (3), 79 (5), 77 (7).

1-Isopropyl-2,3-dimethoxy-5-methylbenzene (**10**): To a solution of diol **2** (14.9 mg, 89.6 µmol) in acetone (5 mL) was added potassium carbonate (131 mg, 948 µmol) and dimethyl sulfate (170 µL, 1.79 mmol). The mixture was stirred at room temperature for 18 h. The mixture was then cooled to 0 °C, quenched with an aqueous solution of sodium hydroxide (1 M, 10 mL) and stirred at room temperature for 3 h. It was then extracted with dichloromethane (3 × 10 mL), washed with a saturated aqueous solution of ammonium chloride (2 × 10 mL), water (2 × 10 mL) and brine (10 mL). The organic layer was dried over anhydrous sodium sulfate, filtered and the solvent was rotary evaporated. Comparison of the NMR data of the crude residue with the literature description [46] confirmed the formation of the expected dimethylation product. 

RI (DB-5/ Wax): 1386/1776; Rf = 0.35 (Hex/AcOEt 19:1); EI-MS (70 eV) *m/z* 194 [M]^+^ (95), 179 (100), 164 (67), 147 (17), 119 (25), 91 (19), 77 (11).

## Figures and Tables

**Table 1:** Composition of the essential oil of the aerial part of *Pentacalia herzogii* from Bolivia.

Peak No.	Compound	RI (DB-5) ^1^	RI (DB-Wax) ^2^	%
3	Hexanal	801	1047	0.0_5_
4	(2*E*)-Hex-2-enal	849	1176	0.0_5_
5	Hexanol	873	1327	tr
6	Nonene	893	923	tr
7	Tricyclene	918	974	0.0_5_
8	α-Thujene	926	1002	0.1
9	α-Pinene	933	998	33.6
10	Camphene	943	1029	0.1
11	β-Pinene	970 ^3^	1068	0.6
12	Sabinene	970 ^3^	1087	1.6
13	Dehydro-1,8-cineole	986	1154	tr
14	Myrcene	992	1136	1.2
15	α-Phellandrene	1001	1128	0.8
16	Δ^3^-Carene	1007	1112	0.1
17	α-Terpinene	1013	1142	tr
18	*para*-Cymene	1021	1229	1.9
19	Limonene	1028 ^3^	1163	21.6
20	β-Phellandrene	1028 ^3^	1167	0.1
21	1,8-Cineole	1028 ^3^	1169	tr
22	(*Z*)-β-Ocimene	1039	1205	tr
23	(*E*)-β-Ocimene	1049	1220	0.0_5_
24	γ-Terpinene	1056	1207	0.3
25	*cis*-Sabinene hydrate	1064	1430	tr
26	Octanol	1075	1524 ^3^	tr
27	Terpinolene	1088 ^3^	1242	0.1
28	*para*-Cymenene	1088 ^3^	1390	tr
29	Linalool	1100	1521	0.0_5_
30	Nonanal	1104	1357	tr
31	(*E*)-4,8-Dimethylnona-1,3,7-triene	1118	1277	0.0_5_
32	*trans*-Limonene oxide	1134	1409	tr
33	Terpinen-4-ol	1174	1555	0.0_5_
34	α-Terpineol	1189	1648	tr
35	Isothymol methyl ether (**9**)	1234	1539 ^3^	6
36	Thymol methyl ether	1239	1552	6.6
37	Carvacrol methyl ether	1246	1560 ^3^	0.6
38	Isomer of thymol methyl ether	1252	1571	tr
39	Carvacrol	1307	2157	tr
40	Herzogole (**1**)	1330	1749	9.2
41	δ-Elemene	1336	1434	0.3
42	α-Cubebene	1348	1421	tr
43	α-Ylangene	1369	1440	tr
44	α-Copaene	1372	1448	0.0_5_
45	α-Isocomene	1380 ^3^	1482	tr
46	β-Bourbonene	1380 ^3^	1473	tr
47	1-Isopropyl-2,3-dimethoxy-5-methylbenzene (**10**)	1387 ^3^	1776 ^3^	tr
48	β-Cubebene	1387 ^3^	1495	0.1
49	β-Elemene	1390	1545 ^3^	tr
50	Opposita-4(15),11-diene	1413 ^3^	1560 ^3^	tr
51	β-Caryophyllene	1413 ^3^	1545 ^3^	0.3
52	Cascarilladiene	1413 ^3^	1524 ^3^	tr
53	β-Copaene	1424	1539 ^3^	0.0_5_
54	2,3,5-Trimethoxy-*para*-cymene?	1430	1776 ^3^	tr
55	Isogermacrene D	1439 ^3^	1588	tr
56	Selina-5,11-diene	1439 ^3^	1566	tr
57	α-Humulene	1448	1613	1.1
58	Germacrene D	1477	1655	7.5
59	Liguloxide epimer	1481	1638	0.2
60	β-Selinene	1483	1661	0.1
61	δ-Selinene	1487	1644	0.5
62	α-Selinene	1491 ^3^	1666	0.0_5_
63	*epi*-Cubebol	1491 ^3^	1842	0.2
64	Bicyclogermacrene	1491 ^3^	1676 ^3^	0.5
65	α-Zingiberene	1495	1678	0.2
66	α-Muurolene	1497	1676 ^3^	0.1
67	γ-Cadinene	1508 ^3^	1703	tr
68	β-Bisabolene	1508 ^3^	1684	0.2
69	Cubebol	1510	1892	0.1
70	Kessane	1515	1714	0.1
71	δ-Cadinene	1521	1706	0.2
72	Bulnesoxide	1522	1727	0.7
73	Selina-4(15),7(11)-diene	1529	1718	0.1
74	Germacrene B	1549	1766 ^3^	0.0_5_
75	Gynurenol	1556		0.0_5_
76	Eudesma-5,7(11)-diene	1557	1766 ^3^	0.2
77	(*E*)-Nerolidol	1564	2006	0.5
78	Germacrene D-4-ol	1570	1995	0.0_5_
79	Gynuradienol	1574		tr
80	Caryophyllene oxide	1576	1911	0.0_5_
81	Cubeban-11-ol	1584	1991	tr
82	Eudesm-5-en-11-ol	1596	2057 ^3^	0.1
83	4,10-*diepi*-Guaiol	1608	2057 ^3^	0.1
84	Eremoligenol	1622	2116 ^3^	0.1
85	Isoguaiol isomer(s)	1636	2116 ^3^	0.6
86	β-Eudesmol	1642	2164	0.1
87	α-Eudesmol	1645	2153	tr
88	α-Cadinol	1648	2172	0.1
89	*ent*-Germacra-4(15),5,10(14)-trien-1-ol	1678	2292	0.0_5_
90	Shyobunol	1681	2256	tr
91	Juniper camphor	1687		tr
92	Neophytadiene	1843		0.05
		Total identified		98.8%

^1^ Retention index (RI) on a non-polar DB-5 capillary column. ^2^ Retention index (RI) on a polar DB-Wax capillary column. ^3^ At least two products have the same elution time. When needed, percentages are obtained on the alternate column or by calculation thereof. tr = traces, less than 0.025%. 0.0_5_ = between 0.025% and 0.05%.

**Table 2:** Essential oils of *Senecio* species containing thymol methyl ether and isomers.

Species	Origin	Key Constituents of Essential Oil (%) ^1^	Reference
*S. belgaumensis*	India	1-Undecanol (19.5) β-caryophyllene (18.9) Τhymol methyl ether (0.4)	[15]
*S. bombayensis*	India	Linalool (26.3) β-Cedrene (14.5) Thymol methyl ether (0.7) Carvacrol methyl ether (1.9)	[16]
*S. platyphyllus*	Turkey	β-Pinene (13) α-pinene (10.5) Δ^3^-Carene (10.4) Thymol methyl ether (0.1)	[17]
*S. rufinervis*	India	Germacrene D (33.7 and 32.9) ^2^ Germacrene A (1.2 and 10.5) ^2^ Thymol methyl ether (0.2 and 0.3) ^2^ Carvacrol methyl ether (0.5 and 0.1) ^2^	[18]
*S. scandens* (leaves)	China	α-Pinene (11.9) Dehydrosabinene (6.2) *o*-Methyl thymol (1.3)	[19]
*S. tenuifolius*	India	(*E*)-β-Farnesene (16.9) γ-Curcumene (16.3) β-Caryophyllene (14.5) Thymol methyl ether (0.1)	[20]
*S. trapezuntinus*	Turkey	(*E*)-β-Farnesene (16.9) β-Selinene (11.8) Thymol methyl ether (0.2)	[21]
*S. vernalis* (roots)	Serbia	β-Humulene (1.1) Thymol methyl ether (0.7) Carvacrol methyl ether (0.7)	[22]

^1^ Thymol methyl ether was also isolated from a South African *S. elegans* aerial parts extract [14]. ^2^ Leaves and roots.

**Table 3:** Gas chromatographic (GC) instrumental parameters used in this study.

Parameter	GC-FID	GC-MS
Instrument	Agilent 7890A GC with two FID detectors	Agilent 7890B GC with 5977B MS
Columns	DB-5 and DB-Wax (Agilent) 10 m × 0.10 mm × 0.10 μm	HP-5MS or DB-WaxUI (Agilent) 30 m × 0.25 mm × 0.25 μm
Carrier gas	H_2_ (0.7 mL/min, constant flow)	He (1.0 mL/min, constant flow)
Oven temperature program	35 °C (1 min) to 250 °C at 9 °C/min	40 °C (2 min) to 270 °C (5 min) at 4 °C/min
Injection	Neat, 0.03 µL; split 300:1	Neat, 0.5 µL; split 200:1 and 50:1 ^1^
Injector inlet temperature	250 °C	250 °C
Detector temperature	250 °C	Source 230 °C; quadrupole 150 °C
Other detector parameters	Air 450 mL/min, H_2_ 40 mL/min, N_2_ 30 mL/min	70 eV; scan rate 0.33 s between 40 and 550 amu

^1^ Duplicate injections with these splits were carried out to better observe major and minor compounds.

## Data Availability

Not applicable.

## Supplementary Materials

The following materials are available online. Fragmentation pattern of herzogole **1**; NMR spectra of herzogole **1**; Enamine certificate of analysis for compound **2**. Figure S1: Fragmentation Pattern of Herzogole **1**; Figure S2: NRM spectra of herzogole **1**; Figure S3: Enamine certificate of analysis.
