# Supplementary material for: Essential Oils from Bolivia. XV. Herzogole, an Original Monoterpene Benzodioxole from an Essential Oil from Pentacalia herzogii (Cabrera) Cuatrec
Source: Molecules. 2021 Sep 23;26(19):5766. doi: 10.3390/molecules26195766 (PMC8510058; doi:10.3390/molecules26195766)
Supplement: Supplementary file 1 [file molecules-26-05766-s001.zip › molecules-1363864-supplementary.pdf]

Supplementary Materials

Essential Oils from Bolivia. XV. Herzogole, an Original Monoterpene Benzodioxole from an Essential oil from *Pentacalia herzogii* (Cabrera) Cuatrec.

Alexis St-Gelais*

Laboratoire Phytochemia, 628, Boulevard du Saguenay Ouest, Saguenay, (Québec) Canada, G7J 1H4

Eliana M. Maldonado[1], Gloria Saavedra[1], Samuel Siles-Alvarado[2]

[1]Centro de Tecnología Agroindustrial, Universidad Mayor de San Simón, Cochabamba, 992, Bolivia

[2]Centro Especializado de Instrumentación Analítica, Universidad Mayor de San Simón, Cochabamba, 992, Bolivia

Jérôme Alsarraf, Guy Collin, André Pichette

Centre de recherche sur la Boréalie (CREB), Laboratoire d'analyse et de séparation des essences végétales (LASEVE), Département des sciences fondamentales, Université du Québec à Chicoutimi, Saguenay, G7H 2B1, QC, Canada,

Table of contents

Figure S1. Fragmentation Pattern of Herzogole **1** 2

Figure S2. NRM spectra of herzogole **1** 4

Figure S3. Enamine certificate of analysis 11

Figure S1. Fragmentation Pattern of Herzogole **1**

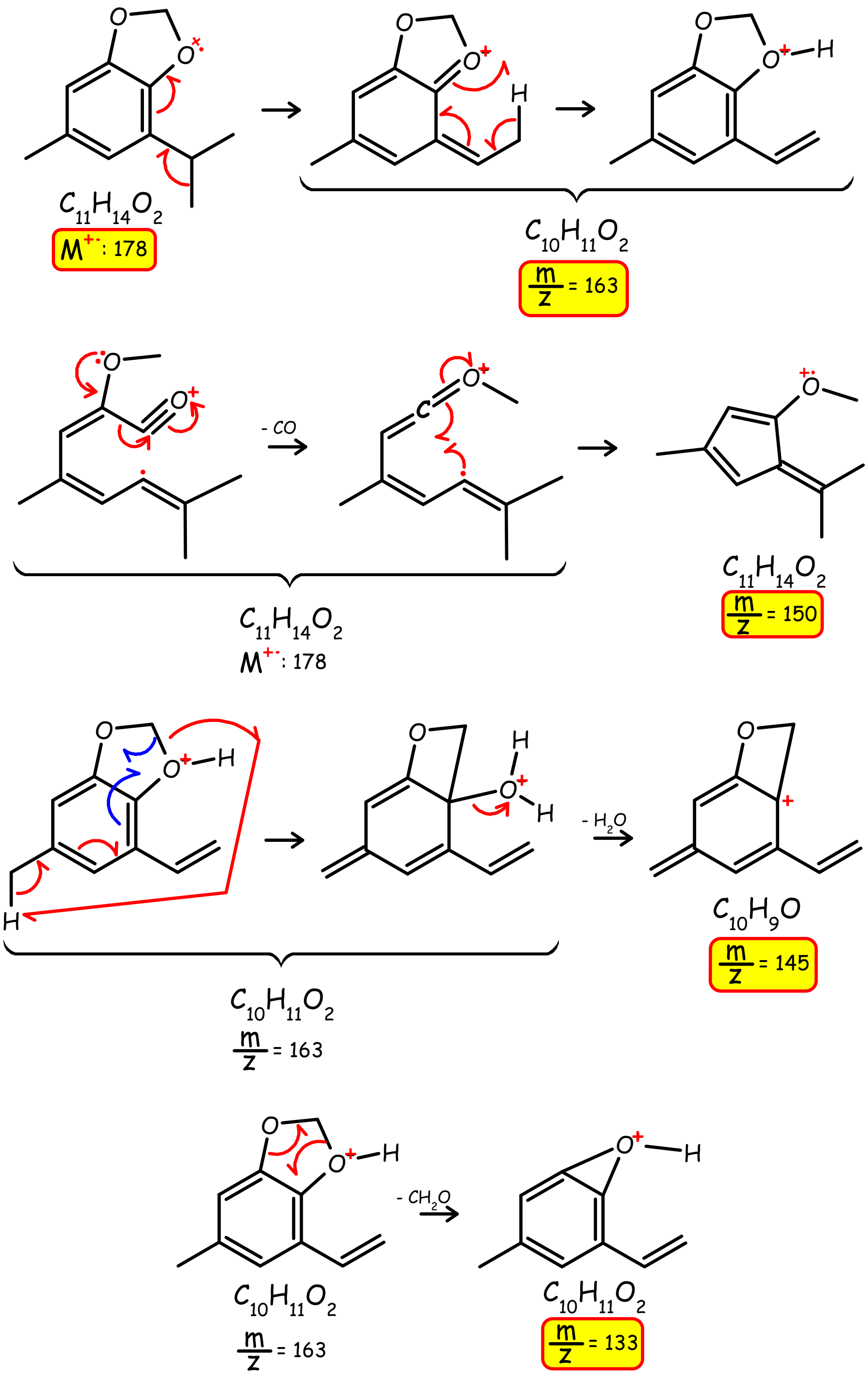

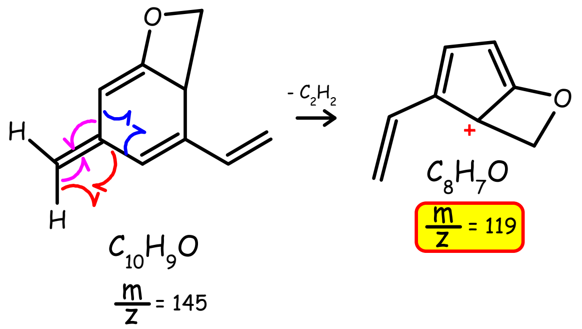
H
H
- C2H2
C8H7O
m/z = 119
C10H9O
m/z = 145

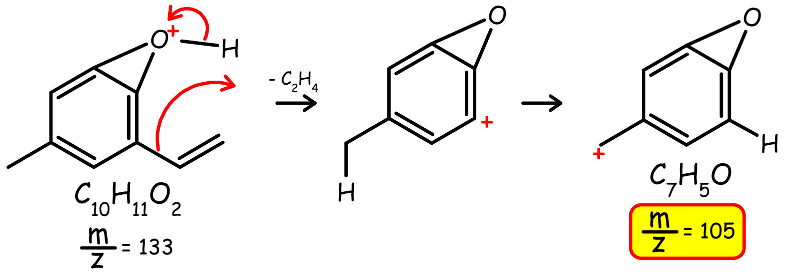
H
- C2H4
H
H
C7H5O
m/z = 105
C10H11O2
m/z = 133

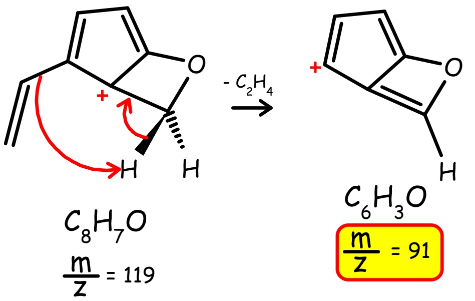
- C2H4
H
H
H
C6H3O
m/z = 91
C8H7O
m/z = 119

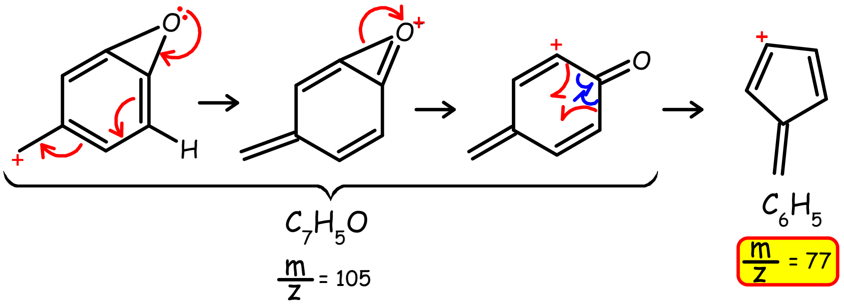
H
C7H5O
m/z = 105
C6H5
m/z = 77

Figure S2. NRM spectra of herzogole **1**

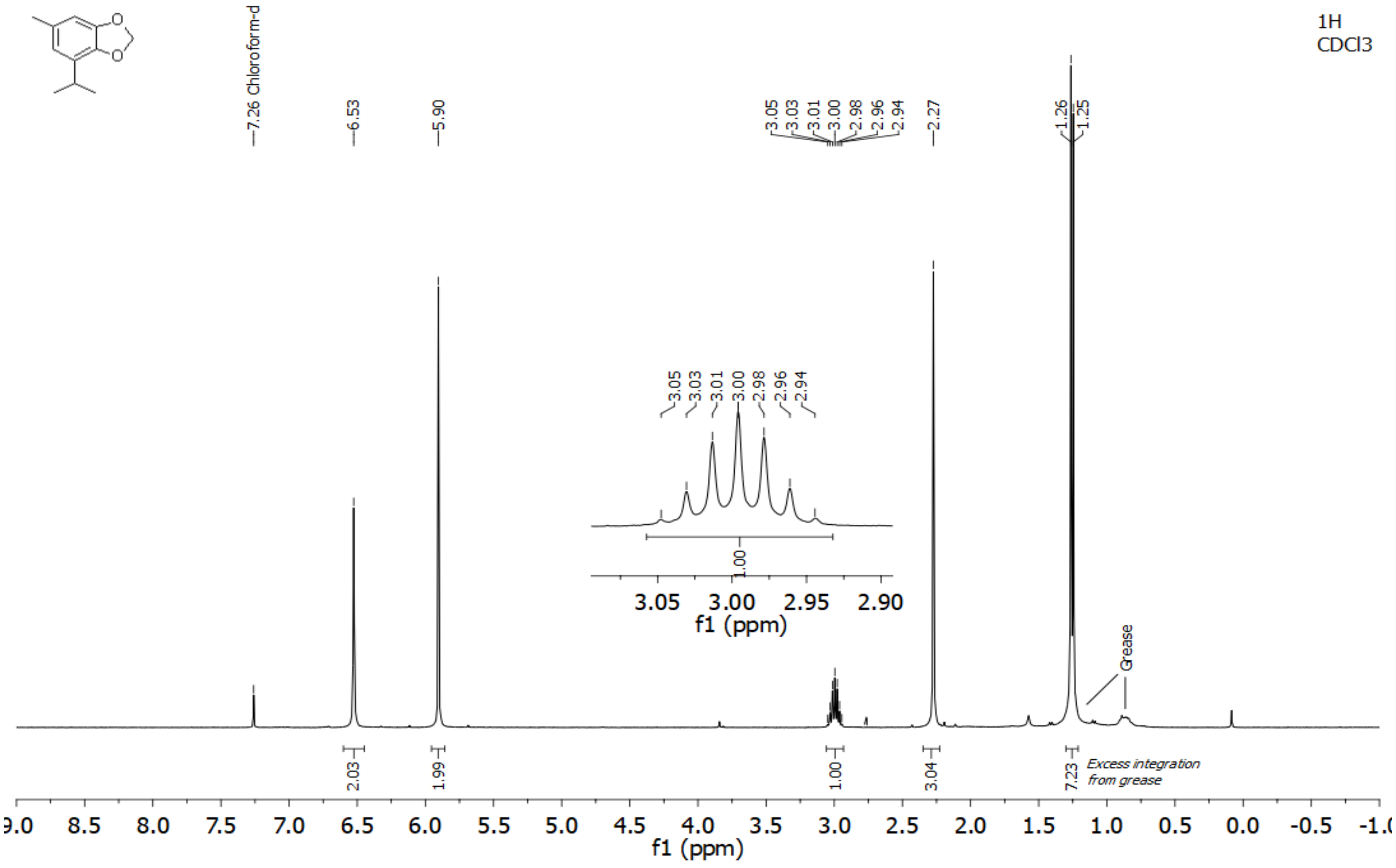

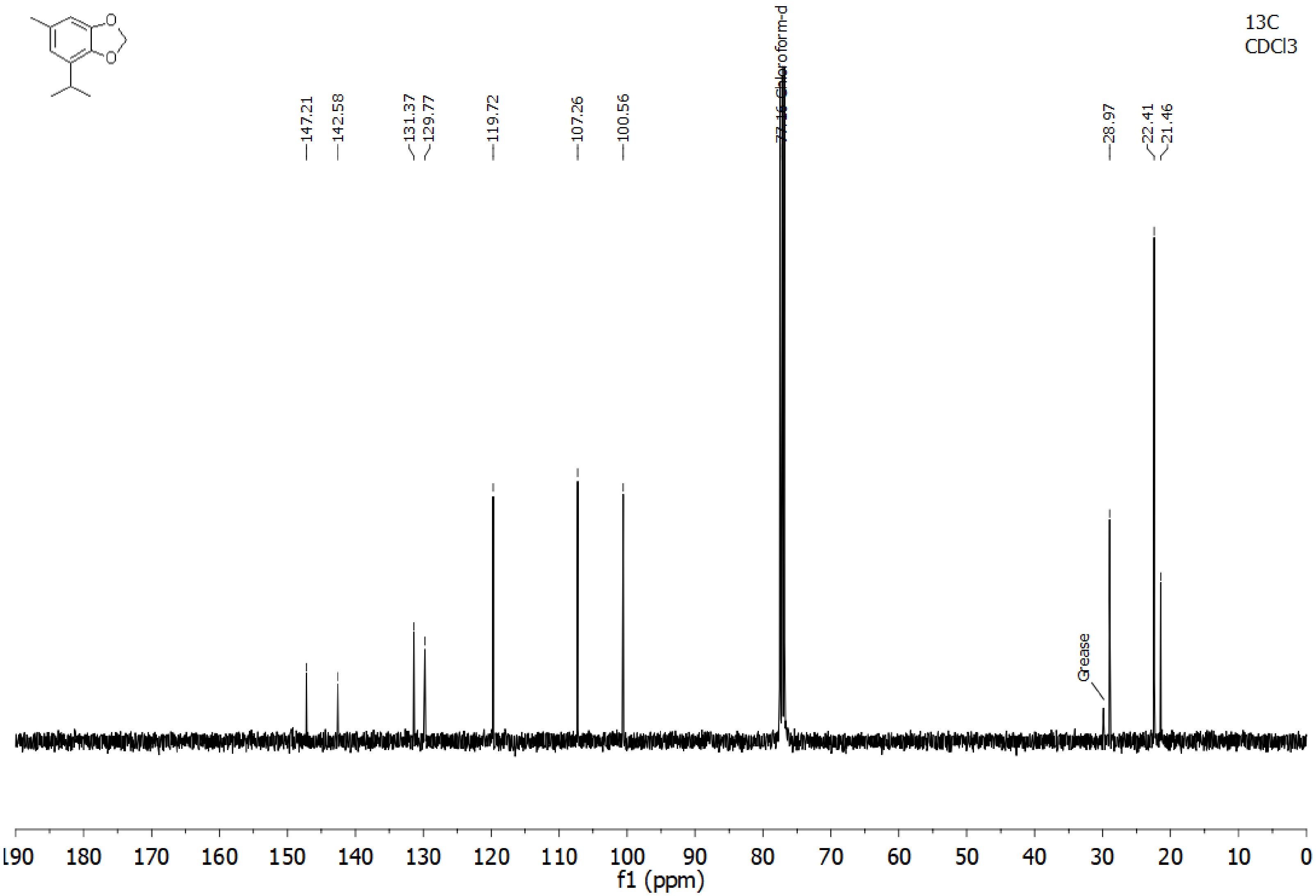
13C
CDCl3
147.21
142.58
131.37
129.77
119.72
107.26
100.56
77.16 Chloroform-d
28.97
22.41
21.46
Grease
190 180 170 160 150 140 130 120 110 100 90 80 70 60 50 40 30 20 10 0
f1 (ppm)

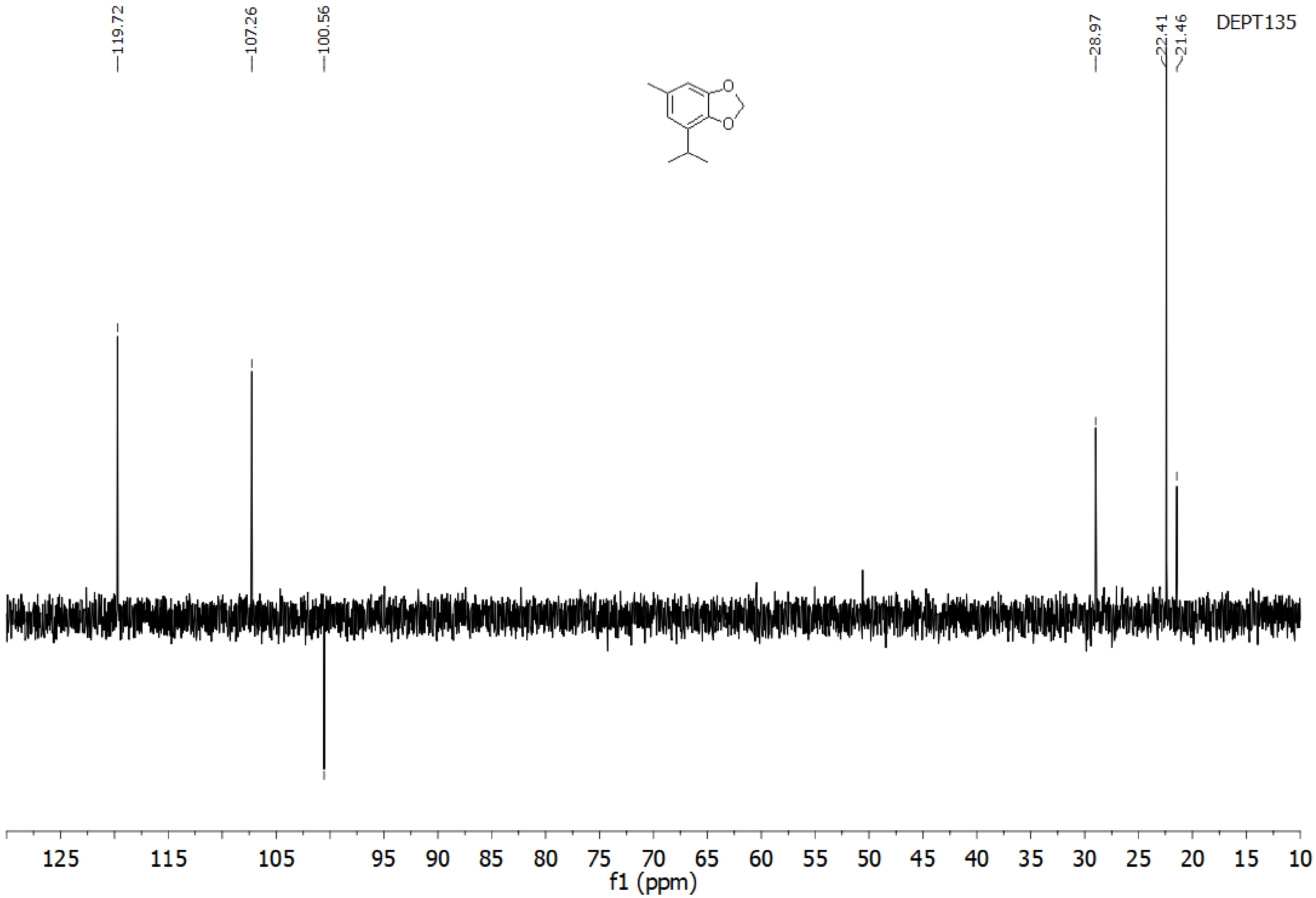
119.72
107.26
100.56
28.97
22.41
21.46
DEPT135
O
O
125 115 105 95 90 85 80 75 70 65 60 55 50 45 40 35 30 25 20 15 10
f1 (ppm)

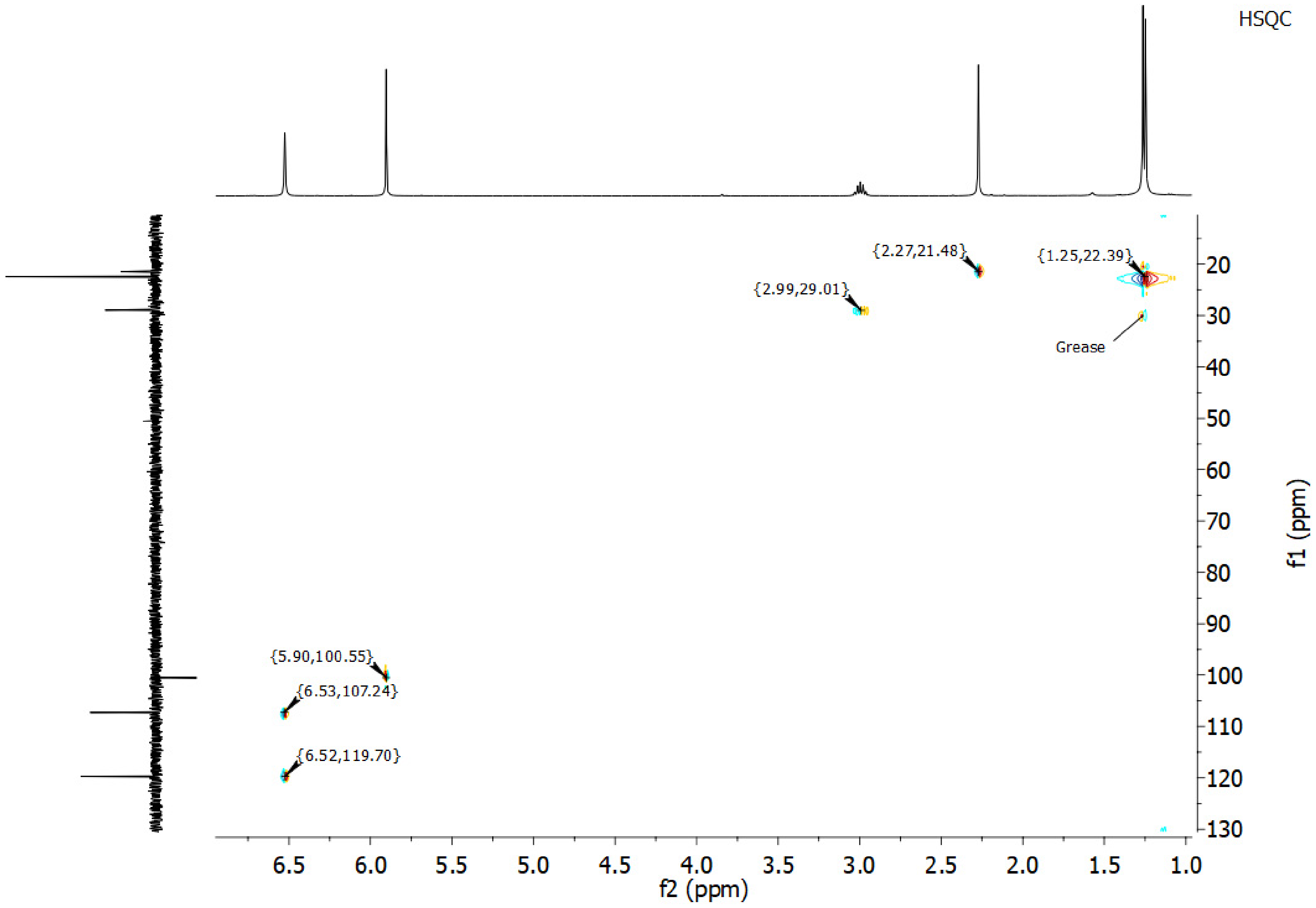
HSQC
{2.27,21.48}
{1.25,22.39}
{2.99,29.01}
Grease
{5.90,100.55}
{6.53,107.24}
{6.52,119.70}
f2 (ppm)
f1 (ppm)

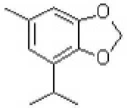

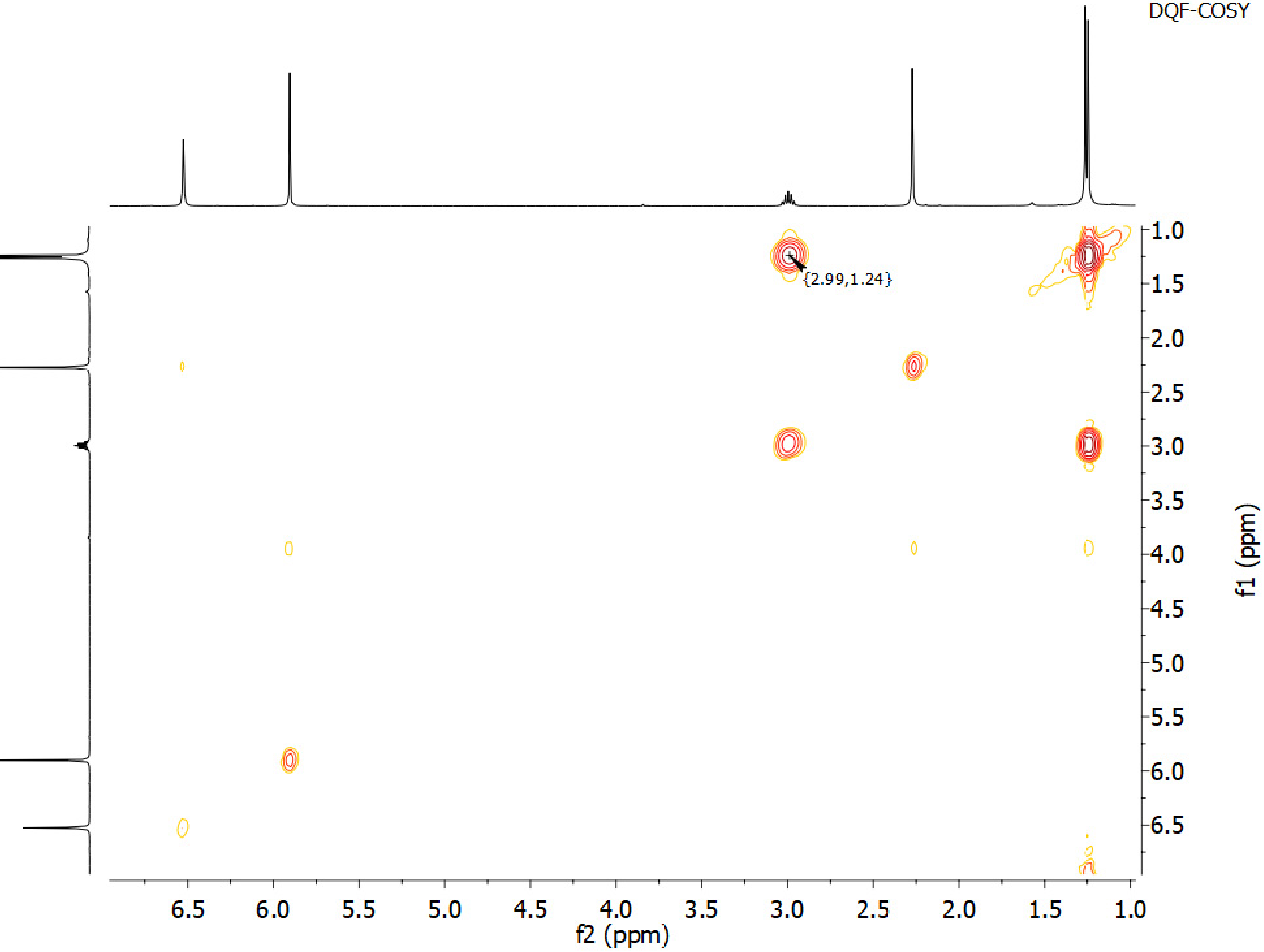
DQF-COSY
{2.99,1.24}
6.5
6.0
5.5
5.0
4.5
4.0
3.5
3.0
2.5
2.0
1.5
1.0
f2 (ppm)
f1 (ppm)

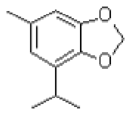

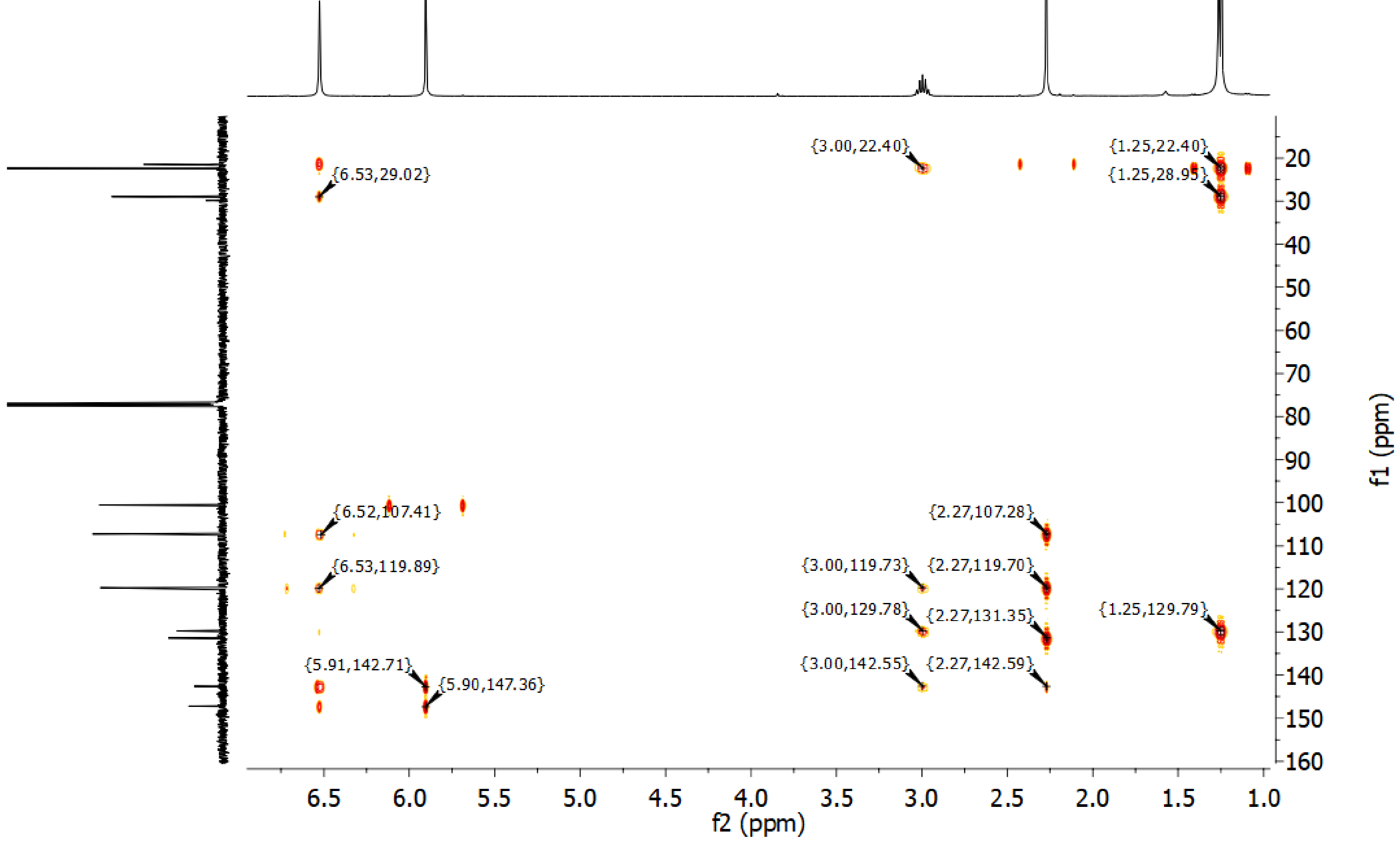
HMBC
{3.00,22.40}
{1.25,22.40}
{6.53,29.02}
{1.25,28.95}
{6.52,107.41}
{2.27,107.28}
{6.53,119.89}
{3.00,119.73}
{2.27,119.70}
{3.00,129.78}
{2.27,131.35}
{1.25,129.79}
{5.91,142.71}
{5.90,147.36}
{3.00,142.55}
{2.27,142.59}
f2 (ppm)
f1 (ppm)

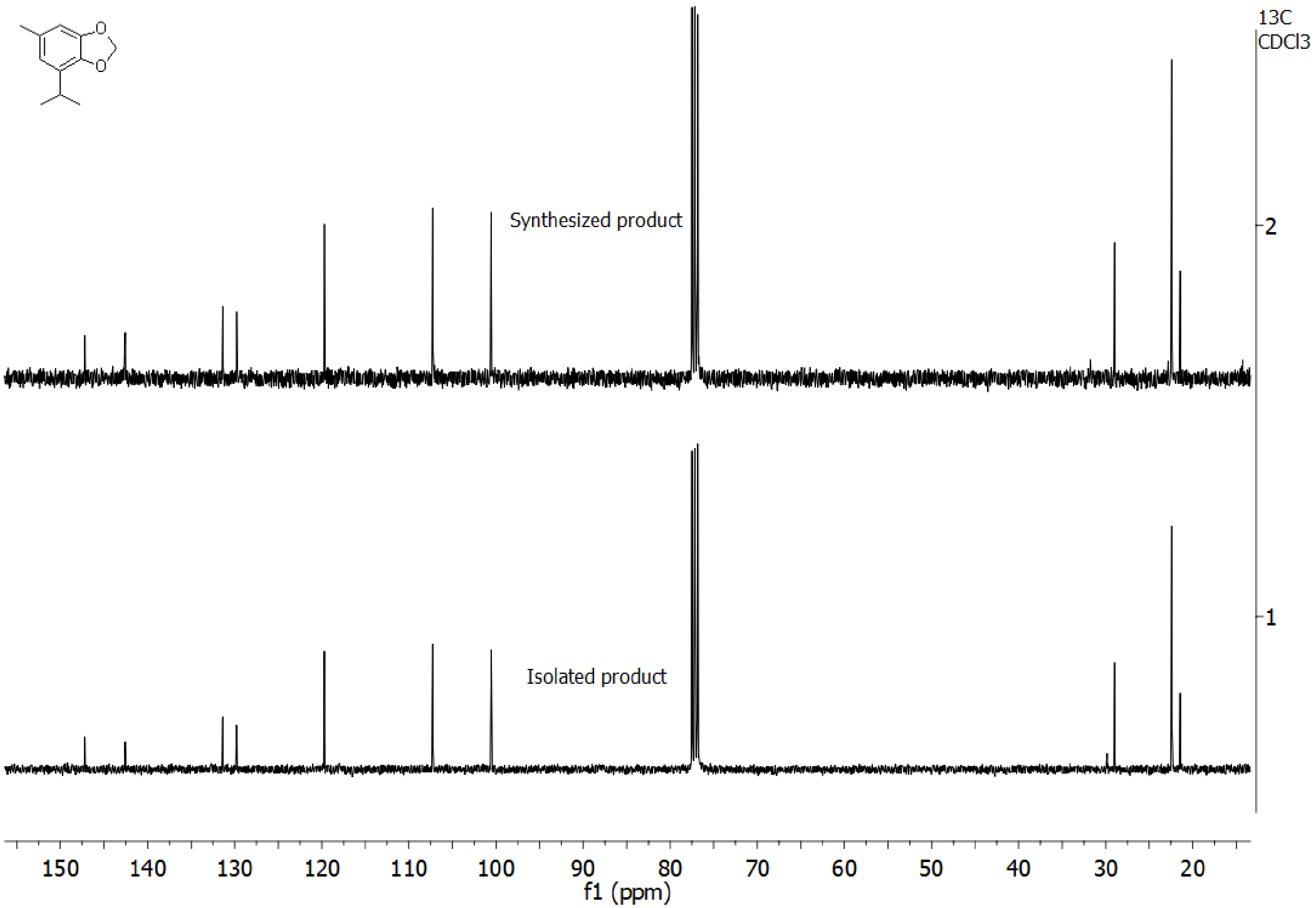
O
O
13C
CDCl3
Synthesized product
2
1
Isolated product
150
140
130
120
110
100
90
80
70
60
50
40
30
20
f1 (ppm)

Figure S3. Enamine certificate of analysis

CERTIFICATE OF ANALYSIS

ENAMINE Ltd.
78 Chervonotkatska street, 02660 Kyiv, Ukraine
Telephone: +380 44 537 32 18
Fax: +380 44 537 32 53
www.enamine.net

Date of release: 2021.03.18
Recommended retest date: 2024.09.21
Sample lot number: 2017-0154152

Description

Name:	5-methyl-3-(propan-2-yl)benzene-1,2-diol
Code(s):	EN300-26990451
Formula:	C10H14O2
Formula weight:	166.217
Storage temperature, °C:	RT
CAS number:	1443106-53-3

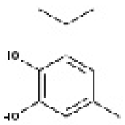

Analysis

Test	Specification	Result
State:	viscous liquid	viscous liquid
Colour:	brown	brown
LCMS	91%	95%,conforms with structure, R907022.PDF

This product is intended for investigational laboratory use only. It is pharmaceutically unrefined, may contain traces of uncharacterized toxic impurities, and is not intended for use in humans. Responsibility for its use and compliance with all applicable laws rests solely with the purchaser.

I hereby certify that data shown in this certificate is correct and accurate.

Dr. A. Konovets,
Head of Quality Control Department

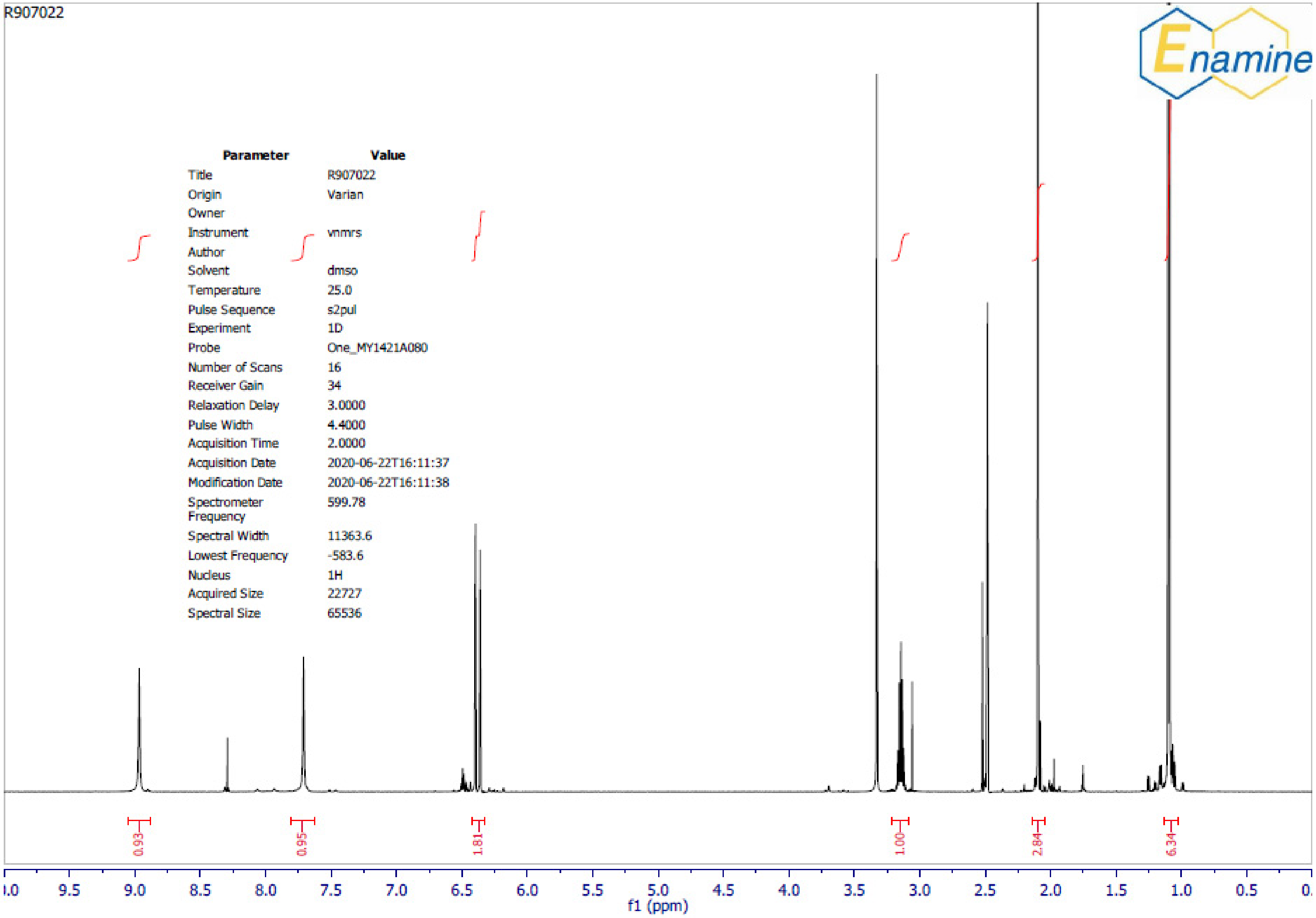
R907022
Parameter Value
Title R907022
Origin Varian
Owner
Instrument vnmrs
Author
Solvent dmso
Temperature 25.0
Pulse Sequence s2pul
Experiment 1D
Probe One_MY1421A080
Number of Scans 16
Receiver Gain 34
Relaxation Delay 3.0000
Pulse Width 4.4000
Acquisition Time 2.0000
Acquisition Date 2020-06-22T16:11:37
Modification Date 2020-06-22T16:11:38
Spectrometer Frequency 599.78
Spectral Width 11363.6
Lowest Frequency -583.6
Nucleus 1H
Acquired Size 22727
Spectral Size 65536
0.93
0.95
1.81
1.00
2.84
6.34
9.5 9.0 8.5 8.0 7.5 7.0 6.5 6.0 5.5 5.0 4.5 4.0 3.5 3.0 2.5 2.0 1.5 1.0 0.5
f1 (ppm)

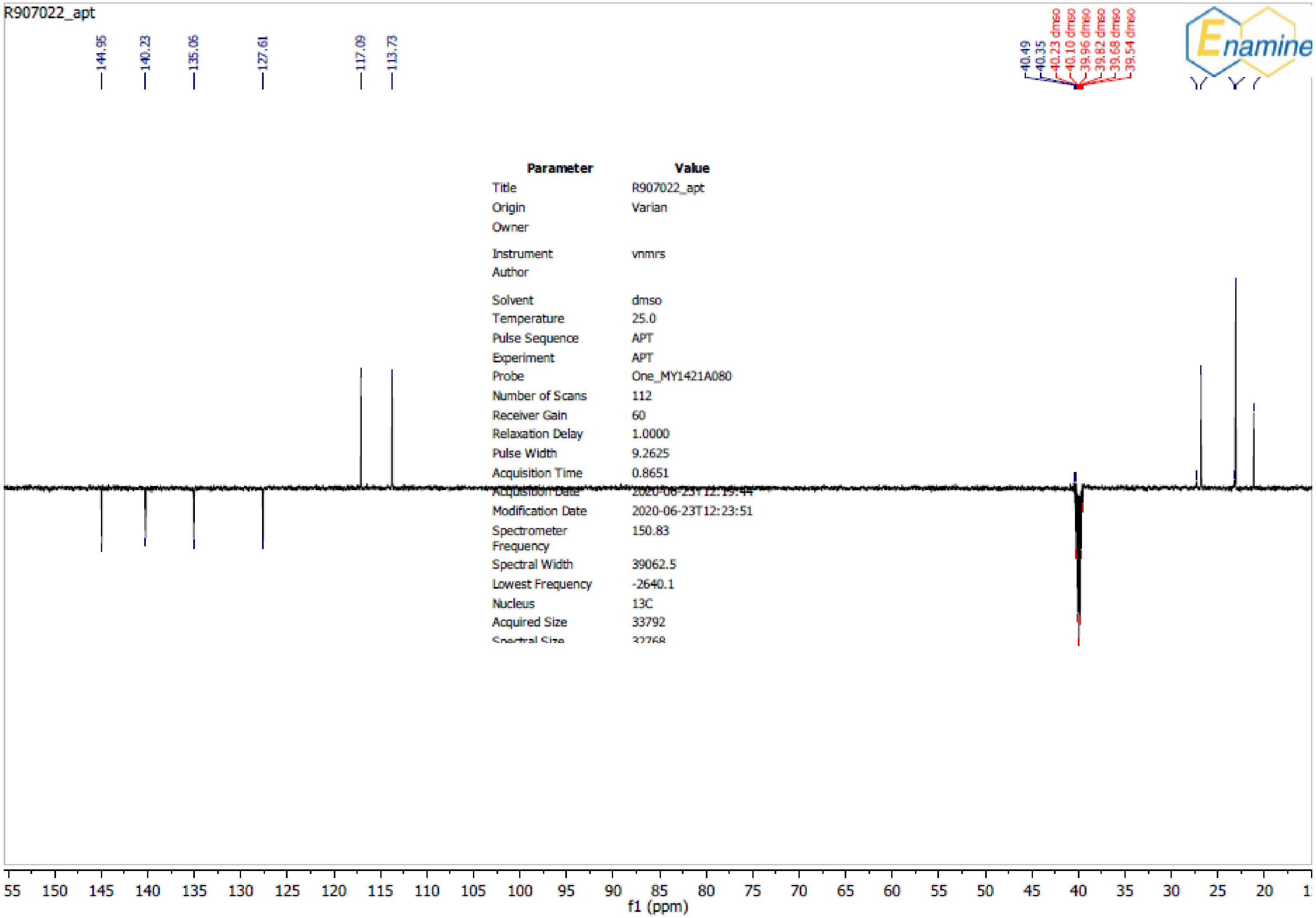
R907022_apt
144.95
140.23
135.06
127.61
117.09
113.73
40.49
40.35
40.23 dmso
40.10 dmso
39.96 dmso
39.82 dmso
39.68 dmso
39.54 dmso
Enamine
Parameter Value
Title R907022_apt
Origin Varian
Owner
Instrument vnmrs
Author
Solvent dmso
Temperature 25.0
Pulse Sequence APT
Experiment APT
Probe One_MY1421A080
Number of Scans 112
Receiver Gain 60
Relaxation Delay 1.0000
Pulse Width 9.2625
Acquisition Time 0.8651
Acquisition Date 2020-06-23T12:19:44
Modification Date 2020-06-23T12:23:51
Spectrometer Frequency 150.83
Spectral Width 39062.5
Lowest Frequency -2640.1
Nucleus 13C
Acquired Size 33792
Spectral Size 32768
55 150 145 140 135 130 125 120 115 110 105 100 95 90 85 80 75 70 65 60 55 50 45 40 35 30 25 20 1
f1 (ppm)
